# Strategies for Heterologous Expression, Synthesis, and Purification of Animal Venom Toxins

**DOI:** 10.3389/fbioe.2021.811905

**Published:** 2022-01-20

**Authors:** Esperanza Rivera-de-Torre, Charlotte Rimbault, Timothy P. Jenkins, Christoffer V. Sørensen, Anna Damsbo, Natalie J. Saez, Yoan Duhoo, Celeste Menuet Hackney, Lars Ellgaard, Andreas H. Laustsen

**Affiliations:** ^1^ Department of Biotechnology and Biomedicine, Technical University of Denmark, Kongens Lyngby, Denmark; ^2^ Institute for Molecular Bioscience, The University of Queensland, St Lucia, QLD, Australia; ^3^ Department of Biology, Linderstrøm-Lang Centre for Protein Science, University of Copenhagen, Copenhagen, Denmark

**Keywords:** animal toxins, venom, neurotoxin, heterologous expression, recombinant toxins, recombinant protein expression, bioinsecticide, toxin-inspired drug

## Abstract

Animal venoms are complex mixtures containing peptides and proteins known as toxins, which are responsible for the deleterious effect of envenomations. Across the animal Kingdom, toxin diversity is enormous, and the ability to understand the biochemical mechanisms governing toxicity is not only relevant for the development of better envenomation therapies, but also for exploiting toxin bioactivities for therapeutic or biotechnological purposes. Most of toxinology research has relied on obtaining the toxins from crude venoms; however, some toxins are difficult to obtain because the venomous animal is endangered, does not thrive in captivity, produces only a small amount of venom, is difficult to milk, or only produces low amounts of the toxin of interest. Heterologous expression of toxins enables the production of sufficient amounts to unlock the biotechnological potential of these bioactive proteins. Moreover, heterologous expression ensures homogeneity, avoids cross-contamination with other venom components, and circumvents the use of crude venom. Heterologous expression is also not only restricted to natural toxins, but allows for the design of toxins with special properties or can take advantage of the increasing amount of transcriptomics and genomics data, enabling the expression of dormant toxin genes. The main challenge when producing toxins is obtaining properly folded proteins with a correct disulfide pattern that ensures the activity of the toxin of interest. This review presents the strategies that can be used to express toxins in bacteria, yeast, insect cells, or mammalian cells, as well as synthetic approaches that do not involve cells, such as cell-free biosynthesis and peptide synthesis. This is accompanied by an overview of the main advantages and drawbacks of these different systems for producing toxins, as well as a discussion of the biosafety considerations that need to be made when working with highly bioactive proteins.

## 1 Introduction

Animal venoms present a treasure trove of biologically active compounds that have evolved to perform highly specialized biochemical tasks, particularly in the contexts of defense against predators and prey capture (Arbuckle et al., 2017; Rivera-de-Torre et al., 2020). These venoms are complex mixtures of peptides and proteins displaying toxic activity, commonly known as toxins, salts, and small metabolites, such as neurotransmitters and nucleosides. To deliver their toxins, animals have evolved different types of piercing structures, such as fangs in snakes, stingers in scorpions, or chelicerae in spiders; by causing physical damage to the skin of prey and perceived predators. Venomous animals deliver their toxins inside the body of their victims, thereby surpassing physical barriers that would normally protect against foreign substances. Moreover, many venoms contain proteolytic enzymes such as metalloproteases, hyaluronidases, and disintegrins that may digest extracellular matrix proteins, causing necrosis to the victim and easing the access of other toxins to their final targets. Other venom components can then compromise cell viability by damaging the cell membrane (e.g., phospholipases A_2_ and pore-forming proteins), affect cell signaling pathways by blocking or activating ion channels (i.e., neurotoxins) (Calvete, 2017; Rivera-de-Torre et al., 2019), or interfere with the blood homeostasis either *via* procoagulant activities (Isbister, 2009) or vasodilatation (Kakumanu et al., 2019). Toxin diversity is thus enormous across the animal kingdom, and it is important to understand the underlying mode of action of medically relevant toxins on their targets in order to devise and evaluate novel therapeutic interventions that serve to neutralize their effects (Salvador et al., 2017; Chinnasamy et al., 2020) or to exploit their bioactivities for therapeutic or biotechnological purposes (Brown and Alewood, 2001; Holford et al., 2018).

To date, most toxin research has relied on sourcing toxins directly from animal venoms (Ahmadi et al., 2020). However, a given toxin represents only a small percentage of the whole venom, which means a low purification yield *via* classic processes such as fractionation. Moreover, the purity of the target toxin is often suboptimal when isolated from whole venoms, complicating subsequent research (Rohou et al., 2007). Toxins are not only scarce and difficult to obtain from the natural source; some toxins are not even present in the venom as they are encoded by dormant genes. Fortunately, the ever-increasing availability of genomic, transcriptomic, and proteomic data from venomous animals has allowed the discovery of dormant or low-expression genes (Palagi et al., 2013; Rivera-de-Torre et al., 2018; Herzig et al., 2020; Walker et al., 2020).

Given the challenges of obtaining rare and low-abundance toxins, other approaches must be taken for procuring animal toxins to fully exploit the potential that lies within their diversity. In this relation, heterologous expression of toxin genes in a laboratory setting presents an exciting and promising alternative to extracting animal toxins from their natural source. This process involves the expression of genes or part of them in a host organism that does not express such genes intrinsically and comes with many advantages. For instance, heterologous expression allows for high yield toxin production while ensuring homogeneity and avoiding cross-contamination with other venom components. Ensuring purity is especially important because toxins are usually part of multigene families, which is why the separation of isoforms by classic chromatographic fractionation might not yield sufficiently pure toxins for particular experiments. Also, heterologous expression strategies minimize the need for animal use in venom research, thereby reducing the risks of accidental envenomations and the stress of animal handling. Thus, heterologous expression also supports the 3Rs in animal research: replacement, reduction, and refinement (Hallen et al., 2007; Valle et al., 2015; Calvete, 2017).

Additionally, the heterologous production of recombinant toxins is not restricted to natural versions of the toxins. The process of expressing toxins heterologously can take advantage of the plethora of molecular biology tools available to design and produce new toxins with unique and desirable properties, which are not present in nature. For example, consensus toxins are artificially designed toxins resembling an average sequence of a collection of natural toxins that might possibly be useful as antigens to obtain broadly neutralizing antibodies that can cross-neutralize multiple native toxin isoforms (de la Rosa et al., 2018, 2019). Moreover, toxins can be modified to modulate their target selectively to induce a therapeutic rather than a harmful toxic effect (Liu et al., 2016; Almeida et al., 2017, 2019).

In this review, we present the possibilities offered by the principal heterologous expression systems (bacteria, yeast, insect cells, and mammalian cells) for the heterologous expression of toxins as well as strategies for producing toxins without cells, such as cell-free biosynthesis or chemical synthesis of peptides. We also discuss the most useful molecular biology features that should be considered to enhance purification and exploit downstream applications. Finally, we highlight some of the most promising research efforts involving toxin expression, e.g., antivenom research, development of bioinsecticides, toxin-derived drug development, and the bioethical considerations surrounding such research activities.

## 2 Classification of Toxins

Designing a successful toxin expression strategy starts with the analysis of the target toxin characteristics. The biochemical and biophysical features of the target toxin may limit the selection of the most appropriate expression host system. Therefore, accurate classification of toxins is key to predict toxin characteristics, as many homologous toxins possess similar biophysical properties.

**FIGURE 1:**
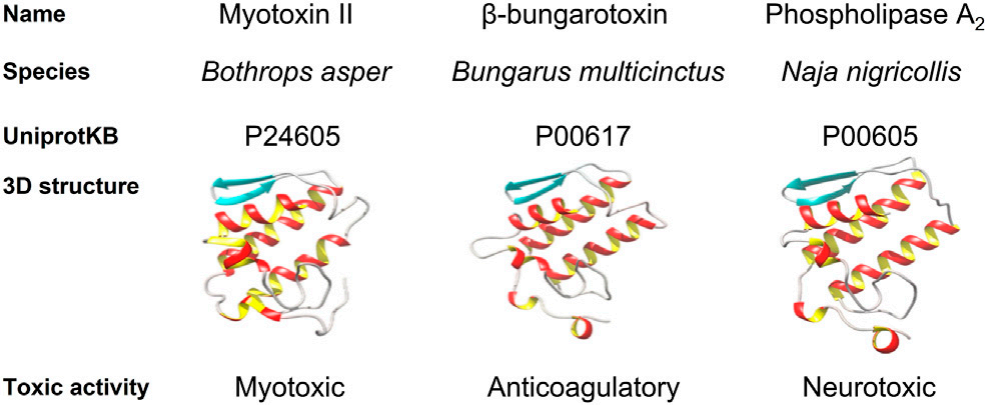
Comparison of Myotoxin II from *Bothrops asper*, β-bungarotoxin from *Bungarus multicinctus* and a phospholipase A_2_ (PLA_2_) from *Naja nigricollis*. They are homologous proteins that cluster together due to sequence similarity and share an archetypical PLA_2_ fold. Nevertheless, they differ in their toxic activity.

Due to the breadth and long history of toxinology, toxin classification has become complex since the most classical categories based on toxic activity coexist with the latest classifications based on protein structure. One of the most basic toxin classifications relies on their ecological role, since toxins serve a distinct purpose and primarily help fulfill three functions: 1) prey capture, 2) defense against predators, and 3) intraspecific competition, for each of which a given toxin has evolved to perform a highly specialized task (Casewell et al., 2013). This abundance of biochemical opportunities has resulted in the enormous diversity of weaponized proteins and peptides that now exist in nature (Casewell et al., 2013). Scientists have categorized toxins based on different variables such as structure similarity and domain homology (Tasoulis and Isbister, 2017). For instance, considering their structural homology, toxins can be grouped in families, including three-finger toxins, cysteine-rich secretory proteins, disintegrins, l-amino acid oxidases, hyaluronidases, metalloproteases, natriuretic peptides, phospholipase A_2_s, C-type lectins, and venom Kunitz-type toxins, to name some. However, one can also group toxins based on their toxic activity, i.e., which physiological system they target (e.g., the cardiovascular, nervous, or immune system), what the specific protein activity is (e.g., myotoxic, neurotoxic, or cardiotoxic), or which pharmacological target they have (e.g., the nicotinic acetylcholine receptor, or voltage-gated sodium/potassium channels) (Fry et al., 2009, 2012; Zhang, 2015). Naturally, structural and functional classifications are interrelated, and some specific folds are directly related to certain toxic activities, e.g., Kunitz-type toxins are usually neurotoxins. However, toxins that cluster together based on structural homology do not necessarily cluster based on function. For instance, while myotoxin II from *Bothrops asper* (P24605), beta-bungarotoxin from *Bungarus multicinctus* (P00617), and PLA_2_ from *Naja nigricollis* (P00605) are all PLA_2_s, they differ widely in their activity. Indeed, P24605 shows myotoxic, P00617 anticoagulatory, and P00605 neurotoxic activity (Figure 1).

**FIGURE 2:**
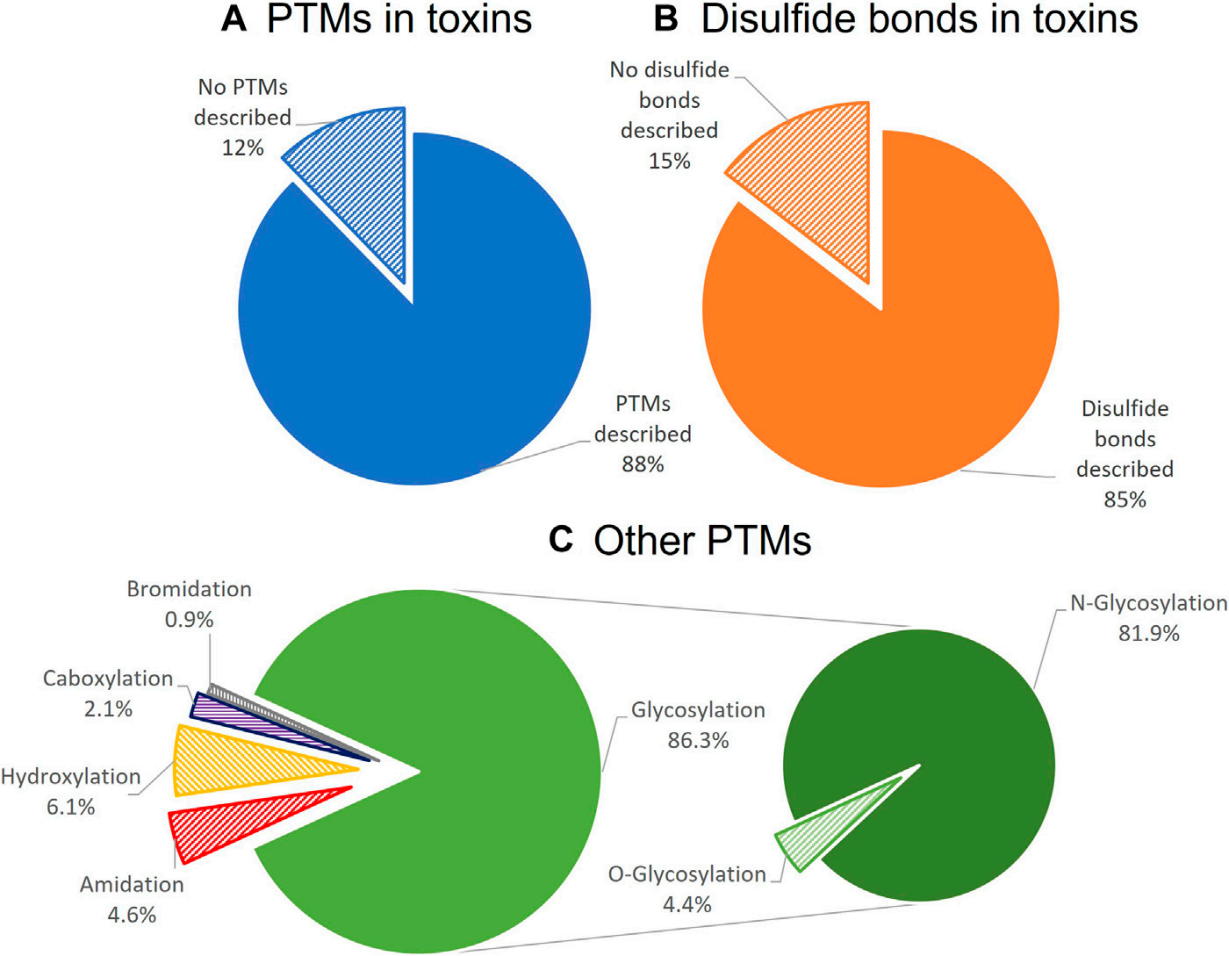
Distribution of PTMs among toxins listed in Uniprot-Toxprot, the Animal Toxin Annotation Project. Only 12% of the listed animal toxins do not have any described PTMs **(A)**. From the toxins with PTMs, 85% had disulfide bonds **(B)**. After disulfide bonds, glycosylation, and especially N-glycosylation, is the most common PTM described for toxins **(C)**.

Finally and especially relevant for heterologous expression, toxins can be classified based on which post-translational modifications (PTMs) they undergo, such as N-glycosylation, O-glycosylation, disulfide-bond formation, methylation, C-terminal amidation, epimerization, bromination, and hydroxylation of proline, amongst others (Walsh et al., 2005; Wood et al., 2009; Degueldre et al., 2017) (Figure 2). Toxins are secreted proteins translated as preproproteins and processed in the endoplasmic reticulum (ER), where a wide variety of PTMs occur as soon as the nascent peptide is exposed to the modifying enzymes. PTMs enable great biochemical diversity of bioactive peptides and often play an essential role in activity, chemical properties, and structural stability. N-glycosylation is one of the most prevalent PTMs in toxins, and the carbohydrate moiety usually has a critical role in toxin stability and solubility. The carbohydrate moiety can also modulate protein functionality and affect enzymatic activity or target recognition (Sandro and Leandro, 2009), as has been described for metalloproteases, three-finger toxins, and serine proteases (Osipov et al., 2004; Silva-Junior et al., 2007; Sandro and Leandro, 2009). For example, the hemorrhagic rhodostoxin from the Malayan pit viper, *Calloselasma rhodostoma,* changes its substrate specificity upon deglycosylation (Tan et al., 1997). Some PTMs have even been shown to occur spontaneously, as was the case for the recombinantly expressed scorpion toxins MeKT11-1 and MeKT11-3, which in aqueous solution underwent cyclization of the N-terminal glutamine, forming pyroglutamate (Kuzmenkov et al., 2018). Even though disulfide bond formation and glycosylation can be achieved in microbial eukaryotic systems, such as yeast, most PTMs need specific enzymatic routes that not all heterologous systems can provide.

An assortment of expression host options is available, ranging from simple bacteria to the most advanced mammalian cell cultures, and choosing the most appropriate expression host for a given toxin can be informed by prior knowledge about the protein. For instance, the structural classification and the possible PTMs provide critical physicochemical information about the target toxin solubility, functionality, stability, and expected yield, narrowing the heterologous expression host options.

## 3 Systems for the Heterologous Expression of Toxins

### 3.1 Bacteria: *Escherichia coli*

Since the first functional recombinant protein was expressed in 1977 (Itakura et al., 1977), bacteria have been the most widely used system for heterologous expression of proteins. Consequently, over time, a whole plethora of tools have been developed to improve bacteria for protein expression in both small and large scales.

**FIGURE 3:**
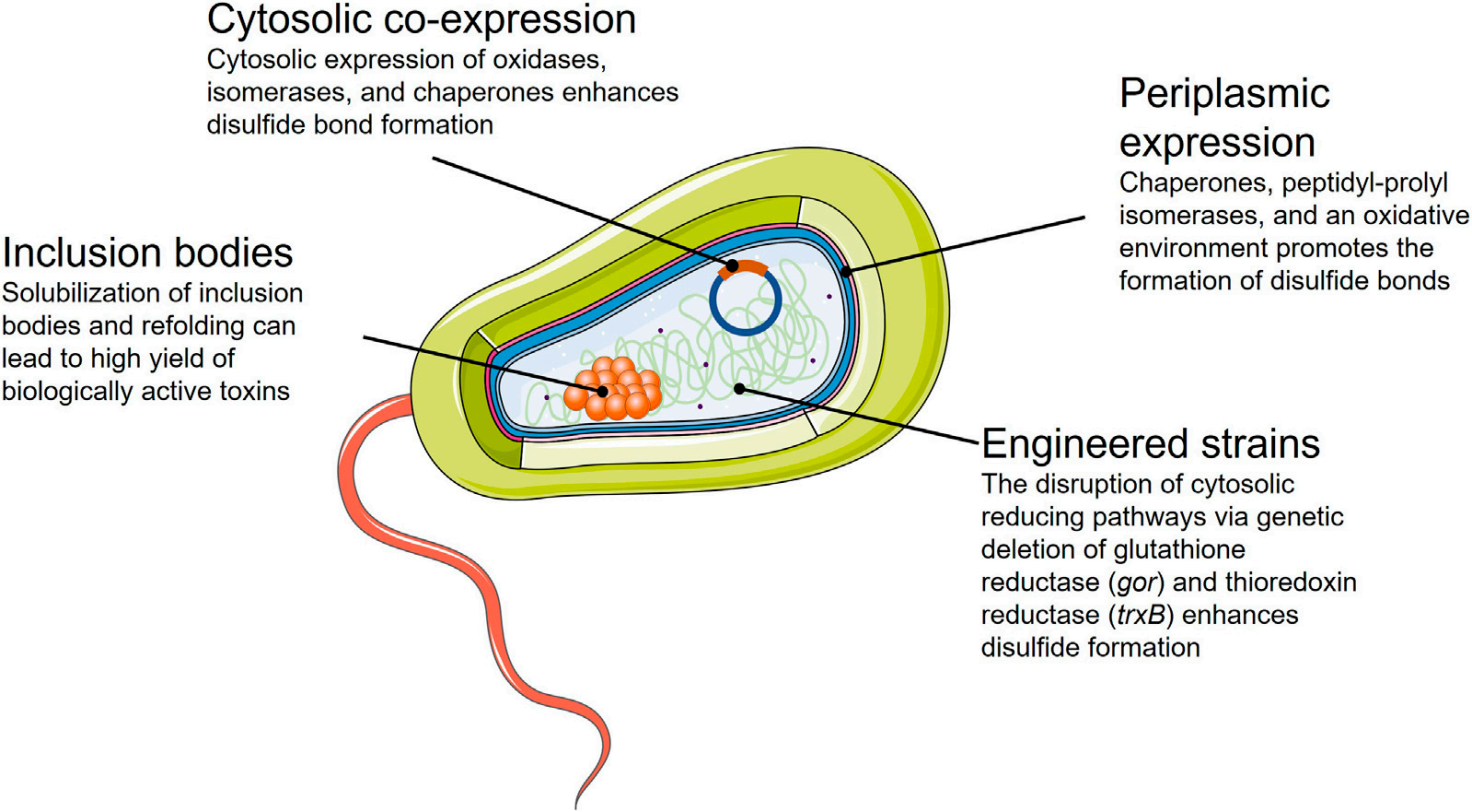
Summary of the strategies available for the successful expression of toxins in an *E. coli* bacterial expression system.

The domain Bacteria comprises a vast number of physiologically and metabolically well-characterized organisms. Fundamental research on bacterial physiology has provisioned a knowledge-based framework to rationally design processes in a sophisticated manner (Carr and Church, 2009; Choe et al., 2016). The acquired knowledge has led to a collection of genetically engineered bacterial chassis for heterologous toxin expression. Among all bacterial hosts used for recombinant protein production, *Escherichia coli* is the most widely utilized. *E. coli* adapts to a large range of physical and chemical culture conditions while accumulating recombinant proteins up to 80% of its dry weight. Using *E. coli*, it is possible to express proteins that are safe to administer as biotherapeutics, with efficient methods to remove endotoxins in place (Mamat et al., 2015; Schneier et al., 2020). Even though bacterial systems possess considerable advantages, the main challenge in producing toxins from eukaryotic organisms in bacteria is the correct formation of disulfide bonds and the incorporation of PTMs that prokaryotic systems cannot introduce. Nevertheless, many genetic tools and techniques exist for expressing recombinant proteins, such as optimized bacterial strains, co-expression with chaperones or foldases, and the use of various promoters for tightly regulated expression (Figure 3).

Animal toxins have been recombinantly expressed in microbial systems since the early 1990s (Boyot et al., 1990; Fiordalisi et al., 1991; Park et al., 1991; Dudler et al., 1992; Kelley et al., 1992). To date, bacterial expression still remains the preferred system for the heterologous expression of toxins, especially for small and cysteine-less toxins like actinoporins from sea anemones (Alegre-Cebollada et al., 2007). Bacterial expression has been successfully used to produce the majority of scorpion toxins produced so far (Amorim et al., 2018), and it has been widely used to express snake toxins (Clement et al., 2016; Shulepko et al., 2017; David et al., 2018; Guerrero-Garzón et al., 2018; Russo et al., 2019), conotoxins from cone snails (Yu et al., 2018; Nielsen et al., 2019; Liu et al., 2020), and spider toxins (Meng et al., 2011; Souza et al., 2012; Chassagnon et al., 2017; Wu et al., 2017). Nevertheless, as mentioned before, PTMs and notably, complex disulfide-bonding patterns pose a considerable challenge for the general use of bacterial expression systems.

Animal venoms are an extremely diverse source of cysteine-rich proteins and peptide-based toxins. Cysteines are usually involved in intramolecular disulfide bridges (Pennington et al., 1999), critical for the structural integrity of the toxins in the extracellular environment, although covalent oligomerization also occurs (Osipov et al., 2004, 2008). Finally, cysteines can also participate in toxin activity *via* disulfide tethering with their target (Gajewiak et al., 2014), further illustrating the critical importance of disulfide bonds in toxins.

Even though a few PTMs can occur spontaneously, by far, most PTMs, including disulfide bonds, require enzymatic catalysis. For example, C-terminal amidation can be critical for toxin function and folding (Benkhadir et al., 2004; Kang et al., 2011), and while prokaryotes do not possess the enzymes for the amidation pathway, this PTM has been successfully introduced in a subsequent biochemical step (Ray et al., 1993). Nevertheless, synthetic production (discussed in Section 5) is usually preferred for production of amidated toxins. Achieving this and other modifications *in vivo* requires co-expression of the responsible enzymes (Du et al., 2019). To further compound this problem, in many cases, the responsible enzymes remain unknown.

Considering the addressed drawbacks of bacterial expression, producing toxins in bacteria might seem suboptimal. However, the simplicity of bacterial expression systems in combination with strategies specially engineered for the production of disulfide-rich proteins can circumvent many of the inherent drawbacks. Additionally, misfolding of target proteins can lead to the formation of inclusion bodies, which might be a beneficial starting point for protein purification.

#### 3.1.1 Strategies for the Expression of Disulfide-Rich Toxins in *E. coli*

##### 3.1.1.1 Periplasmic Expression

A major challenge with intracellular expression of disulfide-rich peptides in *E. coli* is the low yield of correctly folded protein due to the reducing environment of the bacterial cytoplasm. One of the commonly used solutions is to bypass the cytoplasm and have the nascent protein secreted into the periplasm of the bacterium.

In the periplasmic space, correct protein folding is promoted by the presence of chaperones, catalysts of disulfide bond formation, and peptidyl-prolyl isomerases (Goemans et al., 2014). Product secretion to the periplasm occurs when the gene of interest includes a short signal sequence at the N-terminus. The signal sequence directs the precursor proteins to the protein export systems in the cytoplasmic membrane and allows the protein to be translocated across to the periplasmic space. During translocation, the signal sequence is proteolytically removed by signal peptidases, ensuring the N-terminal authenticity of the expressed mature protein (Paetzel et al., 2002).

Periplasmic expression in combination with fusion proteins (see Section 4 below) has been extensively used for the expression of toxins. Sequira et al. described a robust toxin expression method in which thousands of toxins were expressed in the periplasmic space fused to the protein disulfide isomerase DsbC (Sequeira et al., 2017; Turchetto et al., 2017). This strategy has been successfully applied for the expression of myotoxins (Giuliani et al., 2001), Kunitz-type toxins (He et al., 2008), hereunder dendrotoxins from snakes (Smith et al., 1997), conotoxins from cone snails (El Hamdaoui et al., 2019), neurotoxins from spiders (Chow et al., 2020), and beta-defensins from sea anemones (Anangi et al., 2012) to name some.

Unfortunately, signal sequences have unpredictable effects on the production yields of recombinant proteins, and it is not possible to predict how a given signal peptide will perform in combination with a recombinant toxin. Therefore, it is recommended to initially screen signal sequence libraries and check the secretory performance for production of the toxin of interest (Freudl, 2018).

Another related drawback of periplasmic expression is the limited yield of the expressed toxin due to the low throughput capacity of inner membrane transport and the volumetric capacity of the periplasmic compartment. However, it has recently been demonstrated that the harmonization between the target gene expression intensity and the translocon capacity is of importance in the improvement of the production yields for periplasmic expression (Schlegel et al., 2013; Baumgarten et al., 2018). Precise control of the expression intensity of the gene encoding the target protein permits the translocation machinery not to be saturated, and the protein production in the periplasm to be optimized.

##### 3.1.1.2 Engineered Bacterial Strains and Co-Chaperone Expression

Whilst translocation of larger proteins into the periplasm can be inefficient and thus reduce yields, bacterial systems have been developed to allow high yield expression of disulfide-rich proteins within the cytoplasm, such as unique *E. coli* strains. Two genetically engineered *E. coli* strains commercially available are SHuffle^®^ (New England Biolabs) and Origami^™^ (Novagen). These *E. coli* strains promote disulfide formation by disrupting the cytosolic reducing pathways *via* genetic deletion of glutathione reductase (*gor*) and thioredoxin reductase (*trxB*) (Stewart et al., 1998; Bessette et al., 1999) to create a more oxidizing environment. In addition, the SHuffle^®^ strain expresses a periplasmic disulfide isomerase (DsbC) in the cytoplasm to enhance native disulfide-bond formation (Lobstein et al., 2012). SHuffle^®^ and Rosetta-gami™ (an Origami™ derivative) strains have been employed in the recombinant production of venom peptides (Li et al., 2006; Sermadiras et al., 2013; Clement et al., 2016). However, they tend to exhibit low growth and yield (Nozach et al., 2013), and successful expression of correctly folded disulfide-rich peptides can require the co-expression of other chaperones (Levy et al., 2001). This is the case for the CyDisCo system (Nguyen et al., 2011; Gaciarz et al., 2016), characterized by the co-expression of two redox enzymes: the mitochondrial oxidase Erv1p from *Saccharomyces cerevisiae* and human protein-disulfide isomerase (hPDI). Erv1p provides the oxidizing equivalents to generate disulfide bonds *de novo*, and hPDI isomerizes non-native disulfides. The CyDisCo co-expression system has been shown to accommodate highly complex disulfide-bonded proteins (Moilanen et al., 2018). In contrast to the SHuffle and Origami strains, where the reducing pathways are disrupted, the CyDisCo system uses an active enzyme system to improve the formation of disulfide bonds and is highly versatile, as it can function in any *E. coli* strain. This system has also been slightly modified to include co-expression of a conotoxin-specific PDI (csPDI) that was found to significantly accelerate folding of conotoxins *in vitro* (Safavi-Hemami et al., 2016; Nielsen et al., 2019). Recently, the CyDisCo system has been modified to create a more stable version of the system (called DisCoTune), which alleviates potential problems with resource competition (Bertelsen et al., 2021).

#### 3.1.2 Purification From Inclusion Bodies

Despite the multiple molecular biology strategies to express proteins in bacteria that have been presented, expression of recombinant toxins might result in protein aggregates packed into inclusion bodies (IBs). IBs protect *E. coli* from the potential toxicity of the expressed protein, leading to an accumulation that could increase the expression yield. Most protocols are developed and optimized to avoid the appearance of IBs, since it is more straightforward to purify the correctly folded and soluble protein from the periplasm or cytosol. Thus, even though IBs may contain a high percentage of active proteins that can be extracted under non-denaturing conditions (Peternel et al., 2008), the toxins present in IBs are often misfolded and inactive. To alleviate this issue, excellent procedures do, however, exist for the solubilization of inclusion bodies and refolding of their protein content, which consist primarily of the protein of interest. Therefore, several toxins have been expressed in IBs and refolded into structurally stable, bioactive molecules with a high yield and purity (Bayrhuber et al., 2006; Shulepko et al., 2017). Many toxins are remarkably stable and can withstand extreme temperature and pH conditions without denaturation. Therefore, to refold toxins, it is necessary to first disrupt their three-dimensional structure by using chaotropic agents (e.g., guanidinium chloride or urea) that disrupt the hydrogen-bonding network between water molecular solvating the toxin in combination with reducing agents (e.g., dithiothreitol or β-mercaptoethanol), which break incorrectly formed disulfide bonds (Rudolph and Lilie, 1996; Saikia et al., 2021). The refolding process consists of eliminating the denaturating agents through dilution, dialysis, or gel filtration, usually at low temperatures for hours to weeks. Even though it is well known that the protein fold is encoded in its amino acid sequence and the folding process is driven by thermodynamically favored intermediates (Anfinsen, 1973), the specific folding pathway is typically unpredictable. In the case of disulfide-rich toxins, the folding process goes through stable intermediates that require partial unfolding to expose buried non-native disulfide bonds to the action of disulfide reshuffling agents. The disulfide pair reorganization task is catalyzed intracellularly by thiol-disulfide oxidoreductases, e.g., protein disulfide isomerases (PDI). *In vitro*, disulfide isomerization can be achieved using enzymes or redox pairs, such as reduced and oxidized glutathione (GSH/GSSG) in a mildly basic pH environment that promotes the nucleophilic attack of the thiolate anion (Hingorani and Gierasch, 2014; Saikia et al., 2021). Refolding conditions, including temperature, pH, ionic strength, and other specific additives, must be defined and selected on a case-by-case basis, as it is difficult to predict *a priori* (Saikia et al., 2021). Favoring the formation of IBs is not the classical approach but can be worth consideration due to the potential high yield and purity (Hoffmann et al., 2019).

### 3.2 Yeast: *Pichia pastoris*

Prokaryotic expression systems are relatively easy to manipulate and scale up. However, producing proteins from eukaryotic organisms (e.g., animal toxins) in such systems might result in misfolding and lack of PTMs and, as a consequence, result in loss of protein function. Yeast expression systems present an excellent alternative that can address this issue.

Whilst a miscellany of yeast strains exist*, Saccharomyces cerevisiae* and *Pichia pastoris* are the most widely used yeast expression systems. Both can produce disulfide-bonded and glycosylated proteins. However, *P. pastoris* lacks the mannosyl transferase, which yields immunogenic α-1, 3-linked mannosyl terminal linkages in *S. cerevisiae* (Darby et al., 2012) and is, therefore, a preferred system to produce proteins for biotherapeutic purposes.

*P. pastoris* is a methylotrophic yeast commonly used because of its ability to produce proteins in exceptionally high-density cultures. The shuttle vectors used in *P. pastoris* lack episomal status; they are integrated into the *P. pastoris* genome, generating stable and productive strains (Li et al., 2007). This yeast expression system offers a high yield for proteins that, so far, have not been successfully produced in bacteria, such as zinc-metalloproteases from snakes or hyaluronidases from scorpions (Zhu et al., 2010; Jangprasert and Rojnuckarin, 2014; Amorim et al., 2018). For other toxins that can be produced in bacteria, the yield increases when expressed in *P. pastoris*, as seen in the case of the potent blocker of Acid-Sensing Ion Channel 3, the APETx2 from a sea anemone. This protein has a potential application in the treatment of chronic pain, and it has been produced in both bacteria and yeast, showing a four-fold higher yield when produced in *P. pastoris* (Anangi et al., 2012). Snake venom serine proteases and neurotoxins from funnel-web spiders have also been successfully produced in *P. pastoris* in high yield (Pyati et al., 2014; Boldrini-França et al., 2015).

*P. pastoris* only produces low levels of endogenous secretory proteins, making purification of recombinant proteins from the culture media straightforward. Therefore, one of the most popular strategies for protein production in *P. pastoris* involves the fusion of the protein of interest with a signal sequence from *Saccharomyces cerevisiae*: the alpha-mating factor pre-pro signal peptide (α-MF). The α-MF secretion signal consists of two parts: a 19-amino acid N-terminal signal sequence that directs translocation into the ER, followed by a 66-amino acid pro region that mediates receptor-dependent packaging into ER transport vesicles to the extracellular media. However, if the protein of interest that is fused to the α-MF secretion signal folds rapidly in the yeast cytosol, the protein may be unable to cross the ER membrane and enter the secretory pathway. For the expression of toxins in the extracellular media, *P. pastoris* has been used successfully (Anangi et al., 2012; Ahmad et al., 2014; Pyati et al., 2014), although secretion levels have often been variable and dependent on the target protein. There is no golden standard for the secretion of recombinant proteins in *P. pastoris*, and numerous new signal peptides have been found in recent years. As described for periplasmic expression in *E. coli*, signal peptide screening and optimization are necessary to exploit the possibilities for *P. pastoris* expression systems (Aw et al., 2018; Duan et al., 2019).

Although most toxin expression experiments have been performed using the common *P. pastoris* strain X-33 (Guo et al., 2001; Anangi et al., 2010; Zhu et al., 2010; Jangprasert and Rojnuckarin, 2014), some foreign proteins are unstable in *P. pastoris* culture medium due to the action of secreted proteases. To this end, optimizing the culture conditions, such as altering the temperature or pH of the media, or switching to alternative carbon sources, is critical for enhancing yield and reducing toxin degradation. Nevertheless, there are several protease-deficient strains (e.g., SMD1163, SMD1165, and SMD1168) that have been shown effective in reducing degradation (Ahmad et al., 2014; Karbalaei et al., 2020).

As described before, N-glycosylation can have a critical effect on proper protein folding and activity of toxins. However, for therapeutic applications non-human glycosylation patterns are often involved in immunogenic responses that can even lead to anaphylactic shock (Zhou and Qiu, 2019). Therefore, the potential biotherapeutic application of recombinantly expressed toxins necessitates the use of expression systems for which the glycosylation patterns are tolerated by humans. For this purpose, a *P. pastoris* strain, Pichia GlycoSwitch® has been engineered to reproduce “human-like” glycosylation patterns, resulting in reduced immunogenicity of protein products (Karbalaei et al., 2020). Additionally, further modifications, such as PEGylation of toxins produced in yeast, have been demonstrated to lead to toxin products with reduced immunogenicity and extended half-life (Pinheiro-Junior et al., 2021).

**FIGURE 4:**
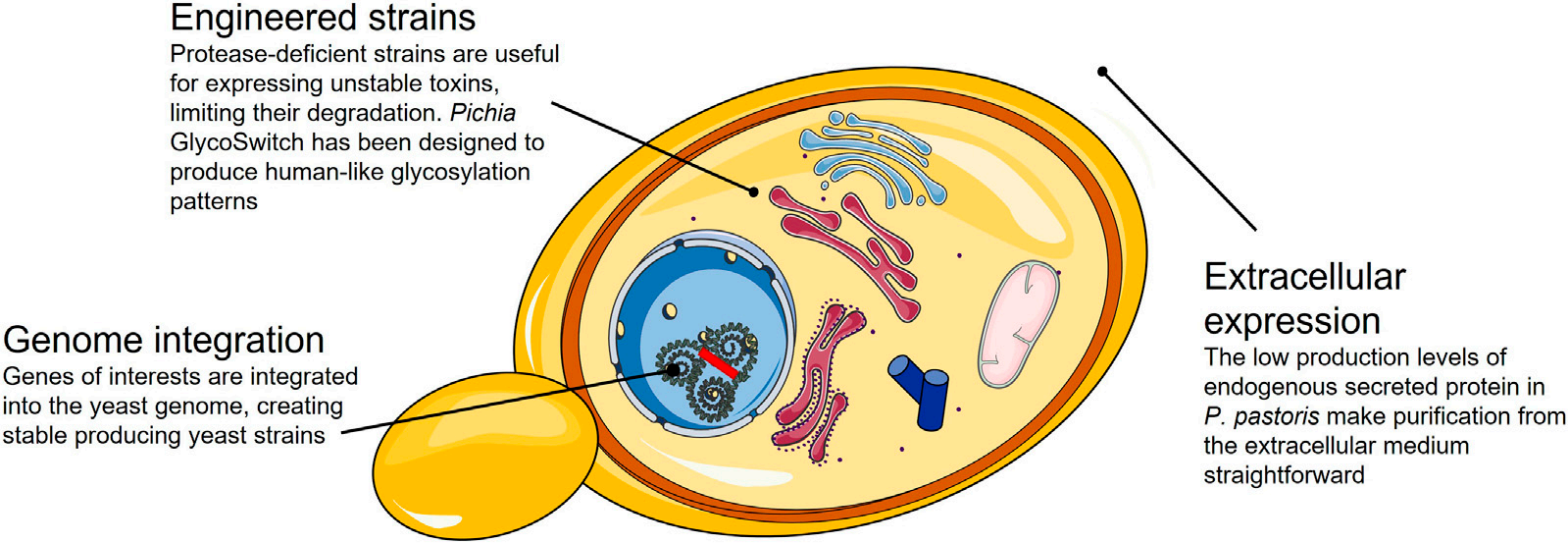
Summary of the strategies available and their advantages for the expression of correctly folded toxins in a *P. pastoris* expression system.

*P. pastoris* is considered an outstanding cell factory for industrial production of recombinant proteins. It is a microbial system relatively easy to scale up in batch/fed-batch systems. Continuous cultivation in bioreactors is also a feasible option with numerous advantages, such as reduction of the running cost and minimization of equipment (Nieto-Taype et al., 2020). Nevertheless, optimization is required to achieve maximum productivity, particularly regarding methanol and sorbitol concentrations, temperature, and incubation times (Karbalaei et al., 2020). Finally, some proteins expressed in *P. pastoris* can be hyper-glycosylated in comparison to their wild-type version, resulting in products with reduced or without biological activity (Figure 4).

### 3.3 Insect Cells: Baculovirus Expression Systems

Insect cell expression systems are an excellent alternative to microbial heterologous expression systems, as these can provide high yield production of functional toxins in a high-throughput format (Hitchman et al., 2012) at a lower cost than mammalian expression systems (López-Vidal et al., 2015). Unlike bacteria, insect cells possess chaperones for correct folding of complex toxins, such as cysteine-rich peptides, and the necessary metabolic routes for complex PTMs, such as glycosylation or acetylation, that do not necessarily exist in microbial systems (Quintero-Hernández et al., 2011). Furthermore, considering the biosynthetic route of toxins produced by arthropods (e.g., arachnids, insects, myriapods), insect cells are often the closest available host system in terms of protein expression processing for animal toxins (Chambers et al., 2018). For example, Pctx1, an inhibitor cystine knot spider toxin, and LALLT, a *Loxosceles* allergen-like toxin have been successfully expressed in insect cells without the need for *in vitro* refolding, unlike bacterial and yeast expression for the same toxin (Escoubas et al., 2003; Justa et al., 2020).

One of the main drawbacks of insect cell expression systems is the complexity of the setup compared to microbial systems in terms of facilities and biochemical tools needed to establish cell lines. However, over the last 30 years, insect cell expression systems have experienced a remarkable evolution, with new versatile and flexible tools and methods being developed (Possee and King, 2016; Chambers et al., 2018).

Insect cell expression systems are presented in various formats that have been extensively used for toxin expression, from whole insect systems, such as silkworm expression systems (Kato et al., 2010), to the most commonly used, cultured insect cell lines, like the baculovirus expression vector system (BEVS) (Deng et al., 2019; Justa et al., 2020; Schemczssen-Graeff et al., 2021). Baculoviruses are a group of viruses that infect insects and are harmless to humans. BEVS is not only a unique system for expressing cysteine-rich toxins, but it has also been useful for testing insecticidal activity of toxin candidates, since the toxicity of an expressed toxin in insect host cells might mean inherent insecticidal activity of the expressed toxin (Justa et al., 2020). BEVS comprises a collection of virus backbones, such as AcMNPV (from *Autograpaha californicata*), OpMNPV (from *Orgyia pseudotsugata*), and BmNVP (from *Bombyx mori*), which can infect various cell lines (Ali et al., 2015). The most commonly used insect cell lines are Sf9 (*Spodoptera frugiperda*) and Hi5 (*Trichoplusia ni*). However, screening other cell lines is recommended since the choice of host cell can impact the expression level, yield, and glycosylation pattern (Geisse, 2007; Wilde et al., 2014).

Toxin expression with BEVS relies on expression promoters for early-stage or late-stage infection (Slack and Arif, 2006). The selection of a promoter impacts the pathology of the baculovirus and can lead to premature cell death due to the effect of the expressed toxin on cell viability (Ardisson-Araújo et al., 2013). Late-stage infection promoters are preferred to obtain high yields of a toxin that affects host viability, such as Ba3 spider toxin (Ardisson-Araújo et al., 2013), as these allow the insect cells to grow enough before producing the toxin that challenges their viability. In comparison, early-stage promoters are not useful for producing the toxin and purifying it for downstream analysis if the toxin has insecticide activity. Early-stage promoters are typically chosen if the goal is to use the toxin as a bioinsecticide since the main objective is to exert toxicity as soon as possible to kill the insect cells.

One of the most attractive features of BEVS is the possibility of having the toxin secreted into the culture media, which allows the establishment of stable expression cell lines expression cell lines. Stable expression cell lines are easy to maintain and attractive for industrial purposes given their high yield and associated product reproducibility. To express a toxin in the insect cell culture media, the target toxin can be fused to the native signal peptide of melittin, a highly expressed bee venom peptide (Vitale et al., 2010). As an example, this strategy has been successfully applied to the expression of α-latrotoxin, a 130 kDa neurotoxin produced by widow spiders that is extremely difficult to extract from the venom gland in large amounts (Volynski et al., 1999).

**FIGURE 5:**
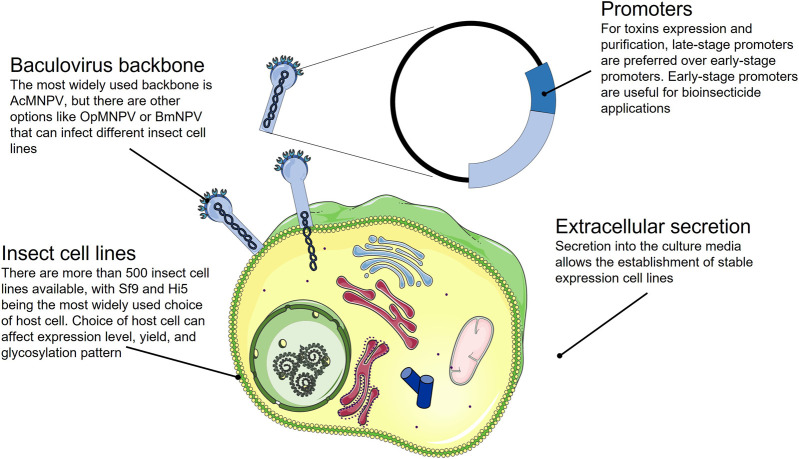
Summary of the strategies available for the expression of correctly folded toxins in a baculovirus insect cell expression system.

Establishing insect cell expression systems usually takes longer than microbial systems. Generating recombinant baculoviruses by conventional methods typically takes up to 6 months. However, new technologies, such as BaculoDirect™ (Thermo Fisher Scientific), can provide faster results, as well as gene editing tools using CRISPR/Cas9 could alleviate some of the cloning difficulties that are often encountered (Pazmiño-Ibarra et al., 2019). The ongoing research on insect cell expression systems focuses on engineering signal peptides and promoters to improve expression, secretion, or folding. For instance, Beek et al. reported an improvement of the lethal activity on insects of the LqhIT2 scorpion toxin by modifying its signal sequence on the AcMNPV virus backbone (van Beek et al., 2003) (Figure 5).

Insect cell expression systems have successfully been used to express animal toxins that cannot be produced or fold properly in microbial systems. Nevertheless, the vast diversity of toxin structures and the requirement to achieve specific PTMs may necessitate the use of even more complex systems, such as mammalian cell lines.

### 3.4 Mammalian Cells

**FIGURE 6:**
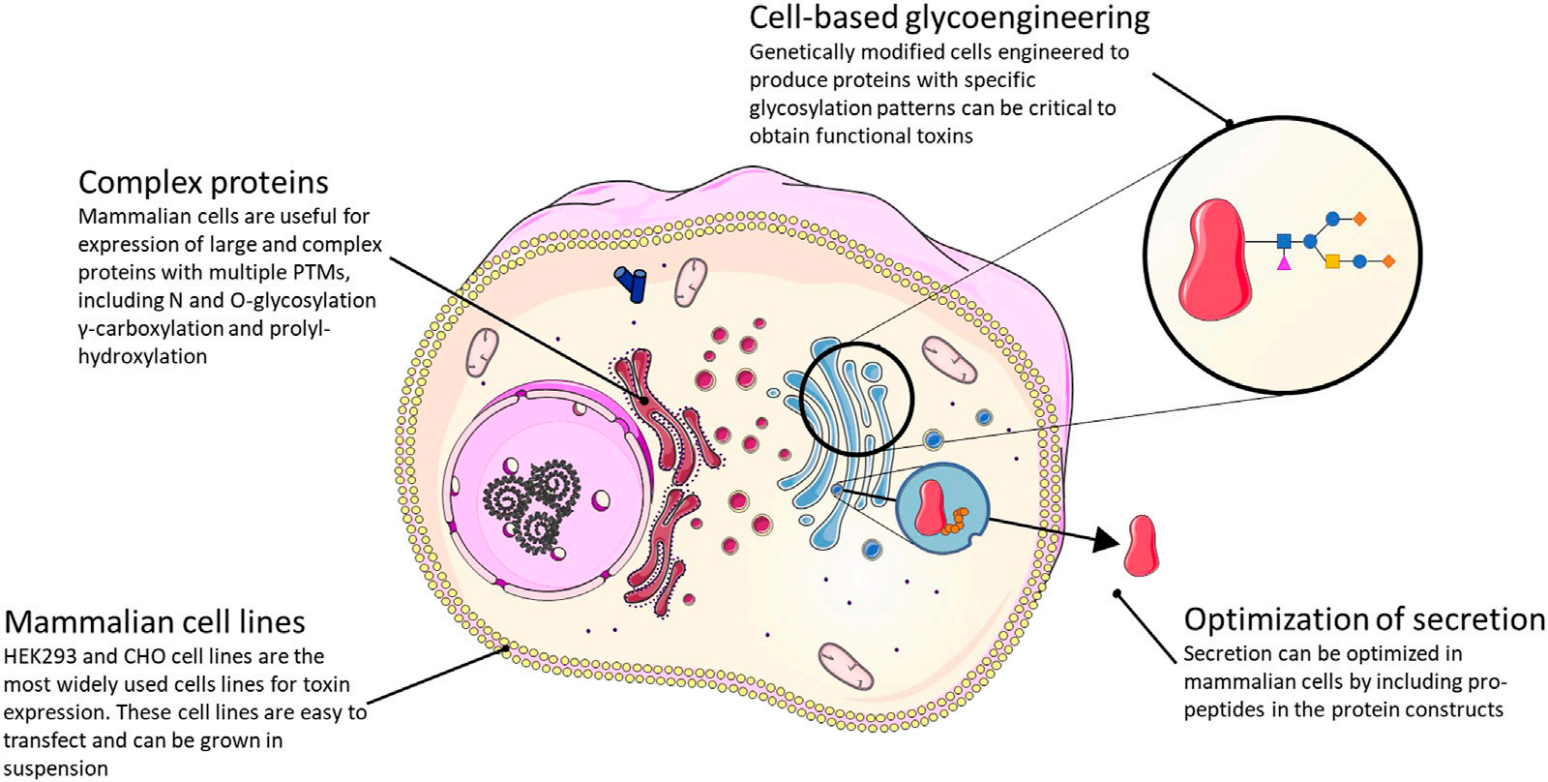
Summary of the strategies available and their main advantages for the expression of functional toxins in a mammalian cell expression system.

Like yeast and insect cells, mammalian cells offer the possibility of producing disulfide-bonded, correctly folded, and post-translationally modified animal toxins. However, compared to the yeast and insect cell systems, mammalian cells are more native-like for many animal toxins. In general, mammalian cells are also likely better suited to produce larger and more complex animal toxins. Mammalian cells are well-developed for recombinant protein expression and are widely used in academia and for industrial production of biopharmaceuticals, such as monoclonal antibodies and disulfide-rich proteins. Among the wide variety of cell lines available, the expression of animal venom toxins has mostly been performed in human embryonic kidney 293 (HEK293) and Chinese hamster ovary (CHO) cells, which are easily transfectable/transducible and can be grown in suspension (Zhu, 2012; Dumont et al., 2016). The main downside of using mammalian cells for protein production is the relatively low yield and high cost due to slow cell growth, laborious culture conditions, and expensive media (Figure 6).

A key area where expression in mammalian cells has a significant potential is for incorporating native(-like) PTMs and disulfide bonds compared to other expression systems. This is desirable in many cases where the addition of specific PTMs influences toxin function and/or stability. Examples include γ-carboxylation of glutamic acid residues and proline hydroxylation. Both modifications are commonly found in conotoxins (Buczek et al., 2005), but cannot be added in native yeast systems since these lack the enzymes necessary to introduce the modifications. Some animal toxins also harbor PTMs that are not, or only rarely, added to mammalian proteins. For instance, different sleep-inducing conotoxins have brominated tryptophan residues (Jimenez et al., 1997, 2004; Buczek et al., 2005). The same modification has been identified in a mammalian brain-specific neuropeptide (Fujii et al., 2002; Tanaka et al., 2003), but the enzyme that performs this modification remains unknown. Thus, recombinant expression of these interesting peptides, which are also γ-carboxylated on several glutamic acid residues, would require further cell line engineering to express the enzyme responsible for the bromotryptophan addition (once identified).

Many animal venom toxins are glycosylated *via* N- and O-glycosylation, which affect folding, trafficking, stability, and function of many, if not most, secretory proteins (Wang et al., 2014b) (Figure 2). In the case of Contulakin-G, an O-glycosylated conotoxin with analgesic properties (Craig et al., 1999), glycosylation positively affects analgesic activity most likely by protecting the peptide from metabolic degradation (Lee et al., 2015). In this and similar cases, cell-based glycoengineering (Ma et al., 2020; Schjoldager et al., 2020), which aims to create cells that express proteins with a specific, desired glycosylation pattern, holds the potential to help produce toxins modified with the functionally relevant glycan structure. Especially in the case of O-glycosylation, which is fundamentally different in yeast (Joshi et al., 2018) and insect cells, mammalian cells offer an advantage. Another use of mammalian cells in recombinant production of animal venom toxins that is largely unexplored is the potential for including propeptides in the protein constructs to optimize secretion. In one known case, the propeptide was demonstrated to be important for efficient secretion of the hydrophobic conotoxin TxVI in the COS7 monkey kidney cell line (Conticello et al., 2003). Of note, secretion of certain animal toxins from mammalian cells may represent a problem in terms of self-intoxication.

Despite the advantages mentioned above, relatively few animal toxins have so far been produced in mammalian cells, compared to the number of animal toxins made in *E. coli*, yeast, and insect cell expression systems. Specific examples include the snake venom proteins rhodocytin (Sasaki et al., 2018) (a heterooctameric C-type lectin with potential as an antiplatelet and antimetastasis biopharmaceutical), acutobin (Wang et al., 2014b) (an α-fibrinogenase with the potential to treat and prevent stroke), ecarin (Jonebring et al., 2012) (a prothrombin activator used, e.g., in diagnostic reagents), and *κ*-bungarotoxin (a neurotoxin used in research on acetylcholine receptors) (Gorman et al., 1997).

The considerable potential of the animal cell expression systems has been convincingly demonstrated by recent work in HEK293 cells, where several hundred “cystine-dense peptides” (CDPs) containing up to 10 cysteines, many of them animal venom toxins, were expressed using a lentivirus transduction system and either displayed on the cell surface or secreted from cells (Correnti et al., 2018; Crook et al., 2018). Moreover, the surface display platform allows for the screening of a large number (tens of thousands) of CDPs (both native sequences and mutagenized variants) for the identification of binding partners of desired targets (Crook et al., 2017, 2018, 2020). This approach has resulted in the identification of one CDP that promotes penetration of the blood-brain barrier by binding the transferrin receptor, and thus shows potential in drug delivery (Crook et al., 2020). The approach has also been utilized to increase accumulation of CDPs in cartilage and was leveraged to deliver a CDP-conjugated steroid, resulting in the alleviation of joint inflammation (Sangar et al., 2020). Taken together, these studies show a large potential for mammalian cells to produce not only native animal venom peptides for characterization, but also to screen mutagenized panels of sequences for identifying interesting novel binding proteins.

**FIGURE 7:**
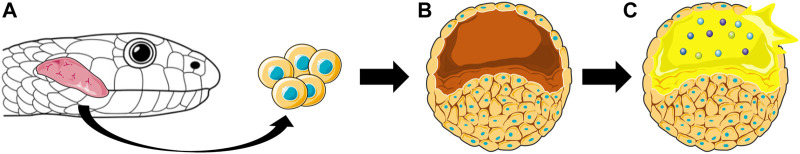
Representation of how venom gland organoids are derived from snake venom gland cells. The cells isolated from the snake venom glands **(A)** are cultured as organoids **(B)** that secrete venom (yellow) containing active toxins (spheres), which can be isolated from the organoids **(C)**.

While mammalian cells will likely become increasingly important for heterologous expression of specific animal venom proteins that cannot be made by other systems, the examples given above demonstrate that the mammalian cell expression systems may require significant engineering to produce completely native structures. Instead, cultured venom gland cells may, for some purposes, constitute the ideal system for the expression of complex, post-translationally modified venom peptides and proteins, although cultured venom gland cells come with the drawback that it may be difficult to isolate a specific single protein/toxin of interest from the complex cocktail produced by such systems. In this respect, the recent demonstration that isolated venom gland cells can be cultured as organoids that secrete active toxins (Post et al., 2020) is highly encouraging. Isolation and immortalization of venom gland cells would allow for the expression of animal toxins in a native environment. Such expression systems could also find application for transcriptional and proteomic characterization of venom proteins to achieve a better understanding of the complex cellular environment, including chaperones and enzymes involved in PTMs, necessary to produce properly folded and modified animal venom toxins (Figure 7). However, while cultured venom gland cells might be beneficial for research applications, it is highly unlikely that they can be used for large-scale manufacture of individual protein products, where monoclonal cell expression systems will be needed.

## 4 Useful Tags and Fusion Proteins for Heterologous Expression and Purification

Tags and fusion proteins are useful molecular tools to facilitate proper protein expression (e.g., enhancing solubility or facilitating disulfide-bond formation) and purification, as well as providing unique features to exploit in downstream applications. These tools significantly differ in their size, ranging from small peptide tags (6–15 amino acids) to large fusion proteins. The smaller tags are mainly used for standardized purification protocols or applications involving the use of commercially available antibodies (i.e., immunofluorescence microscopy, immunoprecipitation, Western blotting), and their versatility and small size generally do not interfere with overall protein structure or function (Kimple et al., 2013). Besides the described functions of tags, fusion proteins offer other specific features, such as enhancing solubility or improving disulfide bond formation.

Since numerous reviews provide a detailed overview of the various different tags and fusion proteins that can be used for recombinant protein expression (Terpe, 2003; Young et al., 2012; Kimple et al., 2013), this review will only focus on the ones that have been extensively used for recombinant expression of toxins.

### 4.1 Tags

The most commonly used affinity tag for protein purification is the poly-His tag, which consists of six to ten consecutive histidine residues. The poly-His tag provides affinity towards divalent metal ions (i.e., Ni^2+^ and Co^2+^), which can be exploited for purification *via* immobilized-metal affinity chromatography (IMAC) (Yang et al., 2003; Bayrhuber et al., 2006; Clement et al., 2016; Shulepko et al., 2017). IMAC resins have a binding capacity of up to 80 mg/ml and tolerate relatively harsh conditions. Additionally, metal binding is largely independent of protein structure, which enables the purification of toxins from IBs under denaturing conditions (see Section 3.1.2). IMAC is also highly suitable for low-cost operation as the resin can be regenerated numerous times. Nevertheless, the poly-His tag charge is critical for binding to the metal ions, therefore restricting the operational pH range for effective purification, which might exclude the utility of the tag for proteins that are not stable at certain pH values (i.e., around the isoelectric point of the protein).

The poly-His tag can readily be utilized when recombinant expression is performed with many different host systems, including bacteria, yeast, mammalian, and baculovirus-infected insect cells. However, the poly-His tag is mostly used in bacterial expression systems, where a single-step purification can lead to relatively pure protein (>80%). On the contrary, the background following his-tag purification is often higher in insect and mammalian cells due to the higher percentage of histidine-rich proteins. Therefore, it is typically necessary to conduct subsequent purification steps. For this reason, other epitope tags like c-Myc and FLAG tags are often employed (Bohlen et al., 2010; Shimokawa-Falcão et al., 2017), for which resins functionalized with specific antibodies are commercially available. Using these alternative tags and columns requires milder binding/elution conditions in comparison with IMAC. Nevertheless, antibody-functionalized resins are more expensive and less stable than Ni^2+^/Co^2+^ functionalized resins used in the purification of His-tagged proteins, making antibody-functionalized resins less attractive from an economic perspective.

### 4.2 Fusion Proteins

The most popular fusion protein used for heterologous expression of toxins is the maltose-binding protein (MBP). MBP is a 42 kDa protein that originates from *E. coli* K12, and, in combination with its native signal peptide, directs protein expression to the periplasmic space of the host. Since MBP is a native protein in *E. coli*, it folds correctly and is soluble when expressed in bacteria, thereby increasing the expression levels and solubility of the fused toxin. MBP has been successfully fused with a variety of different toxins in both bacterial intracellular or periplasmic expression systems (Table 1). Additionally, MBP can be exploited for purification, since columns functionalized with amylose that trap MBP are commercially available. However, amylose resins are gradually degraded by amylase activity present in culture crude extracts, limiting the lifespan of the column.

**TABLE 1:** Summary table of the most widely used fusion proteins for recombinant expression of toxins.

Fusion protein	Size (kDa)	Origin	Used in	Usage	Tested toxins
MBP	42	*E. coli*	Bacteria, yeast, and mammalian cells	Increase solubility and expression.	Snake Smith et al. (1997), Giuliani et al. (2001); He et al. (2008), sea anemone (Anangi et al. (2010), cone snail El Hamdaoui et al. (2019), and scorpion Chow et al. (2020).
				Purification	
GST (glutathione-S-transferase)	26	*Schistosoma japonicum*	*E. coli*	Increase solubility and expression.	Snake Gong et al. (1999); Li et al., 2006; Nozach et al. (2013), bee Zhou et al. (2020), and scorpion Chen et al. (2013)
				Purification	
DsbC	23	*E. coli*	*E.coli*	Increase solubility.	Snakes Nozach et al. (2013); Sequeira et al. (2017); Liu et al. (2021), scorpion, cone snail, and spiders Nozach et al. (2013)
				Promote correct disulfide bond formation	
SUMO	11	Yeast	*E. coli* (kits modified to work in prokaryotes)	Increase solubility and expression.	Spider Souza et al. (2012); Wu et al. (2017), snake Shimokawa-Falcão et al. (2017), and centipede Hou et al. (2013)
Ub19	11	Human	*E. coli*	Increase solubility and expression	Cone snails Nielsen et al. (2019)
TrxA	12	*E. coli*	*E. coli*	Increase solubility. Promote correct disulfide bond formation in *E. coli* periplasm.	Snake Yang et al. (2003); Shulepko et al. (2017); Kaur et al. (2019), and sea anemone Kim et al. (2017)
GFP	27	*Aequorea victoria*	Bacteria, yeast, insect, and mammalian cells	Fluorescent detection	Sea anemone Bakrač et al. (2010), and scorpion Kuzmenkov et al. (2016)

While fusion proteins are often used to solubilize their toxin partner, they can also assist in protein folding and disulfide bond formation. Thioredoxins are oxidoreductases that, through cysteine thiol-disulfide exchange, facilitate the reduction of disulfides, which has been used for expression of snake toxins, as an example (Yang et al., 2003; Shulepko et al., 2017; Kaur et al., 2019). Like MBP, the native *E. coli* thioredoxin A (TrxA) is a highly soluble protein, and when fused with a toxin, TrxA can increase the solubility of the protein construct. Even though TrxA acts as a reductase in the cytosol, it can exert oxidizing activity *in vitro* under the right conditions and thereby promote the formation of disulfide bonds following expression.

Apart from solubility and folding enhancement, fusion proteins can also offer unique characteristics to the toxin. For example, fusion of a toxin with the green fluorescent protein (GFP) makes screening for expression easier and simplifies purification due to its spectroscopic features. Additionally, GFP-tagged toxins can also act as probes for the target of the toxin. This is the case for GFP-equinatoxin, a sea anemone pore-forming protein that recognizes sphingomyelin and is used as a sphingomyelin probe (Bakrač et al., 2010).

Once fusion proteins have fulfilled their mission of improving toxin expression yield, it is often necessary to remove the fusion protein, since it may affect downstream analysis and application. For this purpose, specific protease cleavage sites are often included between the fusion protein and the toxin, such as thrombin and Tobacco etch virus (TEV) cleavage sites (i.e., LVPR|GS and ENLYFQ|G/S, respectively). Both thrombin and TEV cleavage sites leave extra residues after cleavage, which may be undesired depending on the downstream application of the toxin. However, TEV has a broad acceptance of amino acids at position P1, and the TEV cleavage site can be designed so that the residue left after cleavage is the first residue of the native toxin (Sequeira et al., 2017), which makes this cleavage site quite versatile.

Very often, a specific combination of tag and fusion protein is applied during recombinant expression of toxins. For example, as part of the VENOMICS project, Sequiera and others produced thousands of fully oxidized animal venom peptides employing a DsbC fusion partner both for oxidation of disulfides and increased solubility, as well as a His-tag for purification (Sequeira et al., 2017; Turchetto et al., 2017). However, after successful expression and purification, it is important to eliminate both the tag and the fusion protein. This can be a challenging step that reduces the overall process yield dramatically. Therefore, it is necessary to optimize the cleavage conditions to avoid loss of properly folded toxin (e.g., TEV protease is a cysteine protease that requires a specific redox environment to disrupt disulfide bond patterns). After cleavage, it is typically necessary to perform a second purification step based on the molecular weight (i.e., size exclusion chromatography), isoelectric point (i.e., ion-exchange chromatography), hydrophobicity (i.e., reversed-phase HPLC) of the toxin, or even a second affinity purification based on the same tag used for the fusion protein. This second purification often further affects yield.

Given the potential difficulty in eliminating fusion proteins using sequence-dependent cleavage, a clever strategy is to use the small ubiquitin-like modifier (SUMO) as a solubilization tag. SUMO has the advantage of being cleaved by highly specific proteases that recognize the protein structure rather than a particular amino acid sequence (Sermadiras et al., 2013). SUMO originates from yeast, and other eukaryotes have the same conserved family of proteins. Therefore, it is not an optimal tag for expression in these eukaryotic cells due to the presence of intrinsic SUMO proteases. Luckily, commercially available kits for purification of SUMOylated proteins improve the purity of proteins produced in yeast, insects, and mammalian cells.

Finally, another ubiquitin-derived solubility fusion protein widely used in toxin expression is ubiquitin with an internal His-loop (Ub19). Ub19 is an engineered version of the wild-type yeast ubiquitin that presents enhanced solubility and resistance toward nonspecific protease cleavage (Rogov et al., 2012), which takes advantage of the poly-His tag features for purification and detection. Ub19 has been successfully used to express cone snail toxins (Souza et al., 2012; Shimokawa-Falcão et al., 2017; Wu et al., 2017; Nielsen et al., 2019).

The correct selection of fusion proteins in combination with affinity tags is critical for the successful expression and purification of expressed toxins. Unfortunately, given the heterogeneity of structures and physicochemical properties of toxins, there is no winning combination for all cases. It is typically necessary to screen different combinations and potentially explore other expression strategies, such as producing toxins without cells.

## 5 Producing Toxins Without Cells

Depending on the toxin characteristics and the amount necessary for downstream applications, it is worth considering production systems that do not involve cells. Such strategies are restricted to small proteins or peptides; however, the purification protocols are quite straightforward, and it is sometimes faster to achieve a highly pure toxin than with classic heterologous expression.

### 5.1 Cell-Free Proteins Synthesis

**FIGURE 8:**
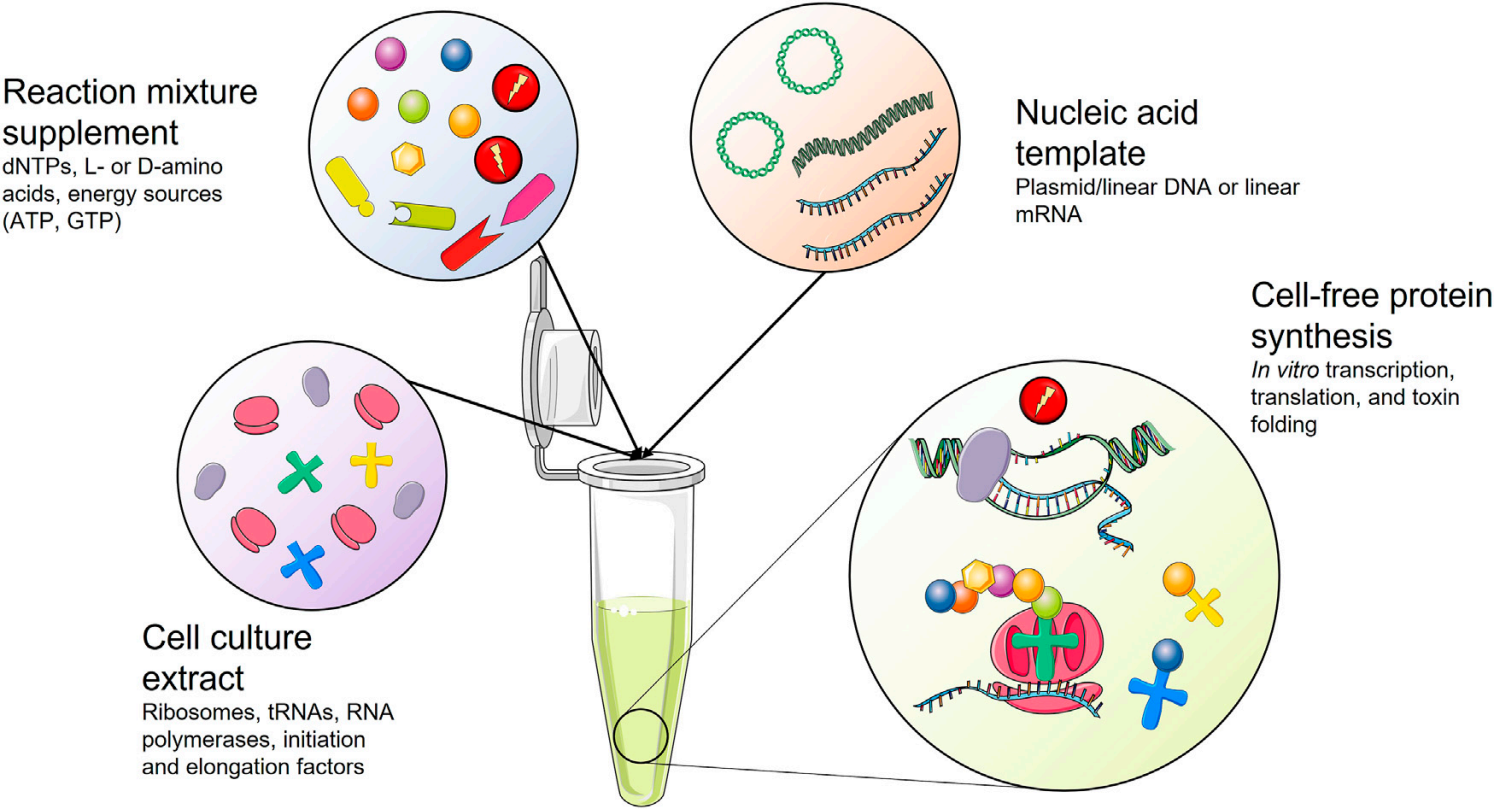
Schematic representation of a cell-free protein synthesis (CFPS) system. Cell culture extract is supplemented with essential reagents for protein synthesis. Upon addition of a nucleic acid template coding for the toxin of interest, the toxin gene is transcribed and translated into a toxin that might need assisted folding.

Cell-free protein synthesis (CFPS) has become increasingly popular for the *in vitro* production of difficult-to-express proteins, such as toxic proteins (Klammt et al., 2006; Zemella et al., 2015). CFPS systems are also suitable for expressing proteins that incorporate non-canonical amino acids or proteins that require tight control of the synthesis in terms of reactant concentrations, which are not easy to regulate in heterologous expression (Zemella et al., 2015; Rolf et al., 2019). These CFPS systems were first developed over 50 years ago to study the genetic code (Ogonah et al., 2017). Briefly, CFPS systems consist of extracts from cultured cells that are treated to reduce the concentration of endogenous RNA and DNA while retaining the minimal machinery for transcription and translation (i.e., RNA polymerases and ribosomes). The culture extracts are supplemented with energy sources (ATP, GTP, etc.) and free amino acids (Sun et al., 2013), and once an enriched extract is in place, expression is initiated by introducing a suitable template, such as linear or circular DNA or mRNA encoding the toxin of interest. Despite the simplicity of CFPS systems, the crude extract source and composition influence the success. Fortunately, many CFPS systems are currently available, originating from Archaea, prokaryotes, fungi, plants, insects, or mammals (Zemella et al., 2015) (Figure 8).

CFPS has one significant advantage for toxin production compared to the use of living cells, which is their tolerability to toxic proteins that would otherwise be problematic to produce in living cells. One example is the expression of a phospholipase A_1_ from *Serratia* sp., which showed extremely low productivity when produced in living cells, but exhibited a 1,000-fold higher yield in a CFPS setup (Lim et al., 2016). Besides dramatically improving the production yield of toxic proteins, CFPS also allows for the synthesis of modified proteins with embedded non-canonical or unnatural amino acids (Oh et al., 2014; Catherine et al., 2015). Several toxins include non-canonical amino acids, such as defensin-like peptide-2 from *Ornithorhynchus anatinus,* which contains a D-Met and has been successfully produced in CFPS (Torres et al., 2005). Furthermore, CFPS often has a simple liquid-handling process and easy scalability, which has allowed for the development of high-throughput protein production systems (Zemella et al., 2015). Although CFPS has many desirable characteristics as an expression platform, it also has notable disadvantages. CFPS systems have low yield compared to heterologous expression systems, and the vulnerability of the nucleic acids encoding the toxins to nucleases present in the culture extracts (resulting in degradation of the DNA/RNA encoding the toxin) makes the establishment of stable production setups difficult (Rolf et al., 2019). The cost of CFPS used to be high in comparison with microbial systems and comparable to mammalian cell expression. However, recent advances in the field and the CFPS high-throughput production have dramatically reduced the cost of the approach, attracting the attention of pharmaceutical companies (Jérôme et al., 2017; Chiba et al., 2021).

Even though CFPS can be used to synthesize many toxins, the wide variety of structures and physicochemical properties necessitates the identification of alternative production systems for more complicated toxins. One of these alternatives is chemical synthesis.

### 5.2 Solid-Phase Peptide Synthesis 

**FIGURE 9:**
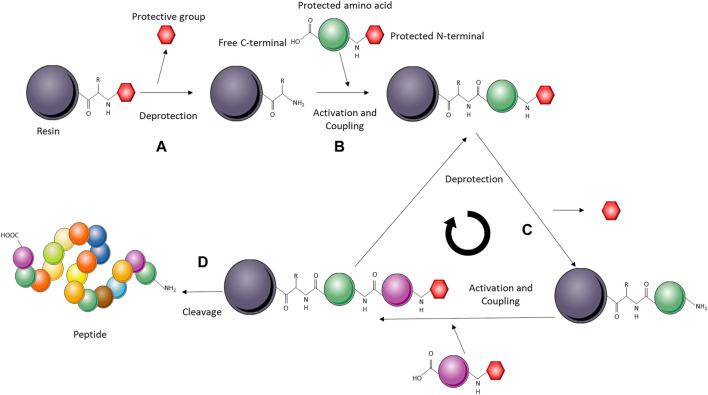
Schematic representation of a solid-phase peptide synthesis (SPPS) system. The solid-phase (resin) is activated for peptide synthesis upon deprotection of the reactive amino group **(A)**. The amino acids are added sequentially on the C-terminal, while remaining protected on the N-terminal. The incoming amino acid forms a peptide bond with the free N-terminal on the resin **(B)**. Deprotection of the amino acid linked to the resin leaves a free amino group ready to react with the next N-protected amino acid **(C)**. The cycle is repeated until all the amino acids are incorporated, upon which the peptide is cleaved from the resin and refolded *in vitro* **(D)**.

Solid-phase peptide synthesis (SPPS) consists of coupling α-amino and side-chain-protected amino acids on a solid support one by one from the C- to the N-terminus (Petrou and Sarigiannis, 2018; Camperi et al., 2020). SPPS has been used to produce toxins that are particularly difficult to express in heterologous systems, such as cysteine-rich peptides (de Araujo et al., 2013; Schroeder et al., 2014; Clement et al., 2016; Jaradat, 2018; Zoukimian et al., 2019). After synthesis, the cysteine-rich peptides can be refolded under oxidative conditions to form the disulfide bonds (Figure 9). Particularly, to achieve the correct disulfide-bond pattern, the cysteines can be protected/deprotected in pairs to obtain correct cysteine-cysteine pairing and, therefore, the biologically active toxin. Peptides synthesized through SPPS are usually no longer than 50 amino acid residues, as longer peptides are difficult to produce in high yield and purity. However, if the toxin of interest is longer than 50 amino acids, techniques, such as segment condensation, can be utilized to combine several peptides (Nuijens et al., 2016; Zuo et al., 2019). Even though the toxin size limitation means that this system cannot be employed for the production of many toxins, SPPS presents indisputable advantages as it is a relatively fast process, and insertions of non-canonical amino acids and several posttranslational modifications are easily incorporated into the toxin *in vitro* (Nuijens et al., 2016; Petrou and Sarigiannis, 2018). Nevertheless, unlike heterologous expression and CFPS, SPPS is more demanding from a technical perspective and might not be feasible to use for classical biochemistry laboratories.

## 6 Applications Derived From Recombinantly Expressed Toxins

Due to their inherent bioactive properties, toxins and toxin derivatives can be used for different types of applications within research, medicine, and industry. The toxin scaffolds themselves can be fine-tuned to bind specific targets of therapeutic or industrial relevance. Toxins might also be coupled to other moieties and used as payloads for advanced biotherapeutics. Finally, the scaffolds can be engineered to lack toxicity, thereby providing safer antigens and immunogens for antibody discovery and immunization. This section will present and discuss the current state-of-the-art within the application of recombinant toxins and toxin derivatives.

### 6.1 Discovery of Broadly-Neutralizing Monoclonal Antibodies Using Designed Consensus Toxins and Cross-Panning on Natural Targets

Recombinant DNA technology allows for expression of toxins that are impossible to obtain from natural sources. It can also prove invaluable when the venomous animal harboring the toxin is rare, does not thrive in captivity, or has very little venom, as exemplified with *Micrurus mipartitus*. This snake produces the lethal toxin mipartoxin, which is not neutralized by existing antivenoms. As this snake venom is challenging to obtain, it is not included in the immunization mixtures used for any existing antivenoms, and the potentially fatal envenomations from this snake cannot be treated (Rey-Suárez et al., 2012). If recombinant mipartoxin could be produced, this toxin could be included as an immunogen together with the venom mixtures used for immunization in traditional antivenom manufacture. In turn, this may lead to a broadening of the neutralization capacity of the antivenom to cover *M. mipartitus* (Bermúdez-Méndez et al., 2018). Recombinant toxins could also be used as antigens for raising monoclonal antibodies or other antibody-like scaffolds (e.g., using phage display technology) (Laustsen, 2018; Jenkins et al., 2019), which likewise could be highly relevant for improving envenomation therapies. Such, monoclonal antibodies could be added to existing antivenoms as fortification agents, or even combined in oligoclonal mixtures to create fully recombinant antivenoms (Kini et al., 2018; Laustsen et al., 2018; Knudsen et al., 2019).

When using heterologous expression, it becomes possible not only to generate the recombinant version of a native toxin but also to generate engineered toxins with special features that improve upon the native toxins for specific purposes, such as consensus toxins that can be used to create polyvalent antivenom. In 2019, de la Rosa et al. demonstrated the utility of a consensus short neurotoxin, which was used to raise a broadly neutralizing serum in both rabbits and horses (de la Rosa et al., 2018, 2019). The researchers showed that this antivenom was able to neutralize venoms from a broad range of elapid snakes from several different continents, thereby demonstrating superior broadly neutralizing effects in comparison with the antibodies obtained from immunization with natural toxins. Whether the broadly neutralizing capacity of these antisera was due to polyclonality or the presence of broadly-neutralizing monoclonal antibodies is not known, but both possibilities exist (Ledsgaard et al., 2018). In the future, it could be speculated that other interesting properties, such as increased immunogenicity and reduced toxicity of toxins used as immunogens for immunization, could be investigated. Also, the construction of modified toxins that are better presented to antibodies in different antibody discovery campaigns could potentially be used to drive binding towards a certain epitope.

**FIGURE 10:**
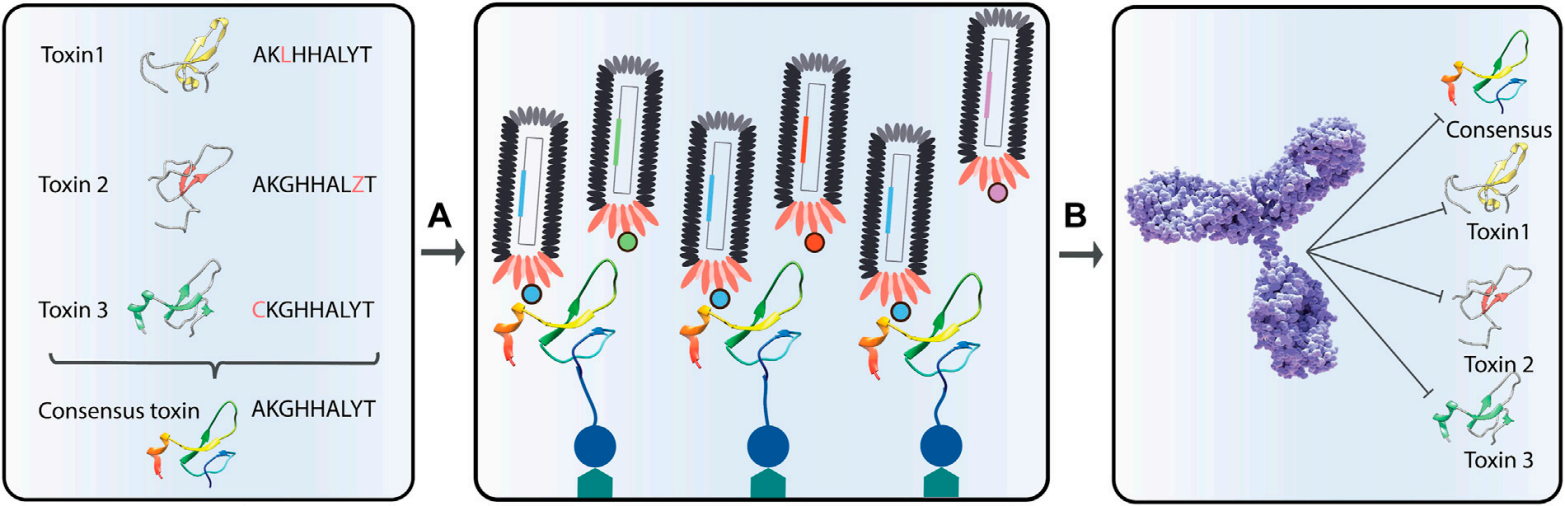
Representation of the discovery of broadly-neutralizing antibodies using consensus toxins. Consensus toxins are designed toxins that represent an average sequence of a collection of homologous toxins **(A)**. By using consensus toxins in phage display selection campaigns **(B)**, selected antibodies might be able to neutralize not only the consensus toxin, but also the natural toxins used in the consensus toxin design.

Finally, the use of heterologous expression systems also allows for expression of toxins without any contaminating toxin isoforms, which can cause trouble in antibody discovery campaigns, as well as create difficulties in the structural and functional characterization of the individual toxins. In both immunization and phage display campaigns, some toxins may dominate and drive the discovery campaign towards antibodies that recognize the contaminating toxin (Lomonte and Calvete, 2017; Laustsen et al., 2018). In immunization processes, this phenomenon is coupled to the immunogenicity of the toxin (Laustsen et al., 2017). In comparison, in phage display experiments, the underlying mechanism for antibody selection is less clear, but speculated to derive from a combination of different toxin properties, such as size and fundamental ability to interact strongly with other proteins through fundamental molecular recognition patterns (Engmark et al., 2016; Krause et al., 2020). For antibody discovery campaigns, where full control of antigen presentation is of high importance, such as when utilizing cross-panning strategies to yield broadly-neutralizing monoclonal antibodies (Ahmadi et al., 2020), recombinant DNA technology for expression of toxins offers great benefits, and it is speculated that many developments and new molecular tools, such as application of tags, consensus toxins, de-immunized toxins, and toxoids, will be brought to life by scientists in the field of toxinology over the next decade (Figure 10).

### 6.2 Bioinsecticides

Control of insect pests is a large concern for agriculture, where pests are reported to cause crop losses in the range of 13–16% (Culliney, 2014). Many insects are also vectors for disease, spreading viruses and parasites among crops, but also to humans and livestock. Unfortunately, such vectors are gaining resistance to traditional chemical insecticides, as has been observed since the 1980s (Brattsten et al., 1986). Due to this unfortunate phenomenon, many traditional insecticides have been de-registered for loss of effectivity or other concerns, such as long-term exposure damage to human and vertebrate health (Windley et al., 2012).

Considering these drawbacks of traditional insecticides, venoms from animals that naturally hunt and feed on insects are a logical source of specific bioinsecticides (Smith et al., 2013). Insecticidal peptides have been discovered in a range of arthropods that prey on insects (Schwartz et al., 2012), including spiders (Bende et al., 2013; King, 2019; Saez and Herzig, 2019), scorpions (Gurevitz et al., 2007; Deng et al., 2019), ants (Touchard et al., 2016; Heep et al., 2019), and centipedes (Yang et al., 2012).

The different requirements for an “ideal” bioinsecticide have been discussed elsewhere (Windley et al., 2012; Saez and Herzig, 2019), but briefly, they should be specific, environmentally benign, have cost-effective high-yield production, and be bioavailable to the insects they target. They need to be specific to insect pest species without being toxic to other animals (e.g., beneficial pollinators) or humans. Consequently, examples of orally active insecticidal toxins are limited but do exist (Mukherjee et al., 2006; Hardy et al., 2013; Guo et al., 2018), and recent studies on whole spider venom reveal that activity upon oral intake by insects is likely to be more common than previously anticipated (Guo et al., 2018).

To improve toxicity upon ingestion of toxin-derived bioinsecticide, delivery strategies to direct toxins to the insect gut, enhancing the insecticidal effects, have been tested by, e.g., fusing peptides to plant lectins or viral coat proteins (Bonning et al., 2014; Herzig et al., 2014; Nakasu et al., 2014; Yang et al., 2014). Another approach consists of delivering insecticidal toxins through the use of transgenic entomopathogens, such as baculoviruses, the *Bacillus thuringiensis* soil bacterium, or the *Metarhizium* fungus. The latter microbes infect insects while simultaneously expressing the insecticidal toxin, thereby showing a synergistic insecticidal effect (Hughes et al., 1997; Wang and St Leger, 2007; Herzig et al., 2014). Entomopathogens are excellent vectors that narrow down the target pest range because of their insect specificity. Moreover, the inherent entomopathogen lethality in combination with the administered bioinsecticide are less likely to cause resistance (Siegwart et al., 2015).

A bioinsecticide derived from the venom of the Blue Mountains funnel-web spider, *Hadronyche versuta*, has already been commercialized as “SPEAR^®^ bioinsecticides” by the company Vestaron by exploiting the broad-spectrum insecticidal activity of the toxin, GS-ω/κ-Hexatoxin-Hv1a (Hv1a). While Hv1a shows insecticidal effect against a range of crop pests, including aphids, spider mites, thrips, whiteflies, and caterpillars, it is safe against honey bees, birds, fish, and humans. Hv1a has also been trialed for malaria control (Bilgo et al., 2017; Lovett et al., 2019), where it was transgenically expressed by a *Metarhizium* entomopathogen with a narrow host range for *Anopheles* mosquitos. A semi-field trial in an endemic malaria region showed that Hv1a-expressing *Metarhizium* outperformed unmodified *Metarhizium* for mosquito eradication (Lovett et al., 2019). Furthermore, the toxic activity of Hv1a in combination with the innate lethality of the entomopathogenic *Metarhizium* acted synergistically, increasing the mosquito susceptibility (Bilgo et al., 2017). While the release of a transgenic *Metarhizium* for malaria control would require further testing and approval, it is a promising biotechnological application of a venom peptide.

Taking advantage of natural toxicity and specificity is an obvious application to exploit animal toxins’ biotechnological potential. Nevertheless, the potential of toxins as tools is not restricted to defeat natural preys. The high binding affinity and target specificity of toxins make them excellent starting points for the development of toxin-inspired biotherapeutics.

### 6.3 Toxin-Inspired Drugs

Toxins whose toxicity relies on modulating mammalian biochemical targets (e.g., blood coagulation cascades, signaling receptors, or ion channels) can be used as valuable leads to develop biotherapeutics. Many toxins have high selectivity and binding affinity to their molecular targets, which can be exploited to develop drugs causing less adverse reactions compared to traditional small molecule drugs. Some limitations of using venom toxins and peptides as drug leads exist, such as limited membrane permeability and therefore reduced bioavailability for humans, as well as poor *in vivo* stability and fast clearance (Otvos and Wade, 2014; Lau and Dunn, 2018). Where native wild-type toxins fall short of the strict activity or selectivity requirements for a drug, “toxineering” approaches (rational engineering of the toxin sequence) may be employed to improve drug properties and minimize off-target activity (Gui et al., 2014; Klint et al., 2015; Neff and Wickenden, 2021), which has already led to several animal-toxin-derived drugs on the market (Table 2).

**TABLE 2:** Examples of approved toxin-derived drugs (Bordon et al., 2020; Herzig et al., 2020).

Drug	Source	Year approved (US FDA)	Indication	Production method	Ref
Captopril^a^	*Bothrops jararaca* (snake)	1981	Antihypertensive	Synthesis	Ondetti et al. (1977)
Batroxobin	*Bothrops sp*. (snake)	Not approved in United States (China: Defibrase, Japan: Reptilase, Korea: Batroxobin; first clinical use early 1990s)	Antithrombotic	Purified from venom and recombinant production	Choi et al. (2018)
Cobratide (cobrotoxin)	*Naja naja atra* (snake)	1998	Painkiller for moderate to severe pain	Purified from venom	Chen and Robinson, (1990)
Eptifibatide	*Sistrurus miliarius* (snake)	1998	Antiplatelet	Synthesis	Ohman et al. (1995)
Tirofiban^a^	*Echis carinatus* (snake)	1998	Antiplatelet	Synthesis	Hartman et al. (1992)
Bivalirudin	*Hirudo medicinalis* (leech)	2000	Anticoagulant	Synthesis	Bates and Weitz (1998)
Enalapril^a^	*Bothrops jararaca* (snake)	2000	Antihypertensive, treatment of diabetic kidney disease, and heart failure	Synthesis	Ferguson et al. (1982)
Desirudin	*Hirudo medicinalis* (leech)	2003	Antithrombotic	Recombinant production	Eriksson et al. (1997)
Ziconotide	*Conus magus* (cone snail)	2004	Painkiller for chronic pain	Synthesis	Sanford, (2013)
Exenatide	*Heloderma suspectum* (lizard)	2005	Treatment of type 2 diabetes	Synthesis	Giannoukakis (2003), Nielsen and Baron (2003)
Lixisenatide	*Heloderma suspectum* (lizard)	2016	Treatment of type 2 diabetes	Synthesis	Christensen et al. (2009), Werner et al. (2010)

a Small molecule.

At first glance, snakes appear to be the most promising source for mammalian-active toxins since many snake species (primarily) prey on mammals. However, early research into therapeutic use of toxins was biased towards snakes due to their large size and the large volumes of venom they produced. Nowadays, mammalian-active toxins with therapeutic potential or toxins active against human pathogens have been found in a range of different animals (Herzig et al., 2020), including, but not limited to, spiders (Saez et al., 2010; Saez and Herzig, 2019), scorpions (Ghosh et al., 2019), centipedes (Hakim et al., 2015; Undheim et al., 2016), and cone snails (Veiseh et al., 2007). In particular, neurotoxins that selectively target the transmitter release machinery, and especially those that affect presynaptic mechanisms by targeting ion channels and receptors, have attracted significant interest from the pharmaceutical fields. These toxins can be used to modulate fundamental processes, such as neurotransmitter release, and may have potential as carriers of molecular cargo and probes (Vetter, 2018; Ovsepian et al., 2019). For example, α-latrotoxin produced by widow spiders and agatoxins from funnel-web spiders are potent neurotoxins affecting presynaptic neurons and are used as molecular probes for studying neurotransmission in mammals and humans (Kaczorowski et al., 2008; Ovsepian et al., 2019).

In recent years, toxins from animal venom have been utilized for novel medical applications. Most notably, chlorotoxin from the venom of the deathstalker scorpion has been engineered as a tool known as “Tumor Paint”. Conjugating the toxin to a fluorescent dye enables high resolution and real-time visualization of solid tumor cancers during surgery (Veiseh et al., 2007). Tumor Paint has shown efficacy in Phase 1 clinical trials in brain, breast, and skin cancers, and is currently undergoing Phase 2/3 clinical trials for pediatric central nervous system tumors (Blaze Bioscience). Chlorotoxin-conjugated graphene oxide has also been used for the selective delivery of doxorubicin, a chemotherapeutic agent, to glioblastoma cells and showed higher efficacy and accumulation of the agent than doxorubicin or graphene oxide-conjugated doxorubicin alone (Wang et al., 2014a). Toxins are also useful for diagnostics, as has been proved for the snake toxin batroxobin (Reptilase^®^), which has been used for decades as a laboratory reagent to measure fibrinogen levels and blood coagulation capability or, RVV-V (Pefakit^®^), derived from a viper toxin capable of activating factor-V of the coagulation cascade, used to diagnose coagulation pathologies (Funk et al., 1971; Schöni et al., 2007; Bordon et al., 2020). Finally, conjugating cytotoxins to tumor-specific antibodies (called immunotoxins) has also enabled specific targeting of the toxins to cancer cells, where the toxins can exert their cytotoxic effects (Russell et al., 2004; Allahyari et al., 2017).

In the last years, several other noteworthy toxin-derived drugs with novel medical applications have entered clinical trials (Bordon et al., 2020). Dalazatide, a synthetic peptide derivative of a toxin from the sun sea anemone (*Stichodactyla helianthus*), which toxicity relies on inhibiting voltage-gated potassium channel Kv1.3, has demonstrated efficacy in the treatment of autoimmune disorders, including psoriasis, arthritis, multiple sclerosis, lupus, and rheumatoid arthritis (Liu et al., 2020). Desmoteplase, a recombinant toxin derivative from vampire bat venom, with a function similar to tissue plasminogen activator, has applications in acute ischemic stroke (Reddrop et al., 2005). Soricidin, a synthetic peptide derived from the venomous saliva of the Northern short-tailed shrew, inhibits transient receptor potential channel TRPV6 and causes selective apoptosis of ovarian and prostate cancer cells (Bowen et al., 2013). Two other molecules, Receptin (RPI-78M) and Pepteron (RPI-MN), are modified toxins (cobratoxin and cobrotoxin, respectively) from cobra venoms, which are active on nicotinic acetylcholine receptors. These are being investigated for efficacy against multiple sclerosis and other neurological disorders (Receptin), human immunodeficiency virus, and herpes simplex virus (Pepteron). Notably, the ability of cobrotoxin, the basis of Pepteron, to inhibit viral replication has also been hypothesized to be useful in the treatment of COVID-19 (Lin et al., 2020). Many other animal venom toxins have shown efficacy in *in vivo* models for a range of important human diseases (Chassagnon et al., 2017; Richards et al., 2018; Anand et al., 2019), suggesting that the future of venom-derived therapeutics may be bright.

## 7 Biosafety Considerations

Working with concentrated solutions or lyophilized preparations of toxins always requires careful handling according to a biosafety plan that follows local regulations. However, with the (low) amounts most often utilized in research labs, working with animal venom toxins presents only a minimal risk to laboratory personnel as well as the public. While their recombinant production allows for the generation of large amounts of single animal toxins, this does not *a priori* present special biosafety issues. In fact, it is worth remembering that working with crude venom can constitute a larger risk due to the combined effect of the individual toxin components in the complex mixture that crude venom represents. In this relation, it is worth mentioning that hypersensitivity reactions have been reported for researchers that have been exposed to lyophilyzed venoms. Therefore, venom powder should be handled under fume hoods, and protective clothing should include face masks to reduce the risk of exposure (Prescott and Potter, 2005; de Medeiros et al., 2008; Chippaux, 2010).

While the high toxicity of certain animal venom toxins, in principle, would allow their use for nefarious purposes, we are not aware of any such reported instances. Still, the European Union [Europe Council Regulation (EC) No. 428/2009] and Australia (Australian Government Federal Register of Legislation Defence and Strategic Goods List 2019) include (all) conotoxins on their lists of regulated toxins, imposing restrictions on their use and export. Apparently, no other animal venom toxins are regulated by any other country (Bjørn-Yoshimoto et al., 2020). Although the United States (CDC, Federal Select Agent Program) and Denmark (Center for Biosikring og Bioberedskab) have removed all conotoxins, except for a small group of paralytic α-conotoxins from their lists of regulated toxins, we strongly support the argument recently put forward that limiting the use of even the most potent animal venom toxins will have little consequence for their possible use as bioweapons (Bjørn-Yoshimoto et al., 2020). Firstly, the misuse of animal venom toxins in bioterrorism seems unrealistic given the fact that much deadlier and more easily available compounds exist. Secondly, strict regulatory measures on the production and use of animal venom toxins in research labs come with the risk of setting back efforts to deliver on the many promises held by these toxins as biopharmaceuticals and research tools. However, we naturally still advice that researchers working with toxins ensure proper safety measures to protect both themselves and the environment, and that proper safety assessments are done on a case-by-case basis.

## 8 Outlook

Animal toxins constitute an excellent source of bioactive compounds with promising biotechnological potentials. However, to be of utility, they must be producible in sufficient quantities not only for research and development efforts but also for later industrial application. In this regard, heterologous expression opens the door to a wide variety of applications, allowing for the development of novel biotechnological tools, such as bioinsecticides or biotherapeutics. As an example, heterologous expression allows for the production of thousands of toxins, which can be screened for interesting bioactivity in a high-throughput setup. Having such expression and purification workflows in place, ideally in an automated fashion, can also allow for the identification of new targets for previously uncharacterized toxins (Sequeira et al., 2017; Duhoo et al., 2019; Reynaud et al., 2020).

There is no single expression and purification strategy that can be applied to all animal toxins, and the chosen workflow will depend on the physicochemical features of the toxin, as well as the desired yield, scale, and downstream application. The production approaches discussed in this review not only enable researchers to produce larger quantities of toxins than can be extracted from natural sources, but also make it possible to work with toxins that are unavailable when the natural source cannot be held in captivity, when the toxin of interest only exists in trace amount within the venom, or when the toxin is not even present in the venom (i.e., dormant genes). Here, heterologous expression makes it possible to exploit the increasing amount of proteomic and transcriptomic data from venomous animals. The heterologous expression and synthetic approaches discussed in this review differ in their capabilities to produce correctly folded and functional toxins with correct PTMs, which should be taken into consideration when selecting the method of toxin production. From the industrial perspective, expression systems present differences in the cost of manufacture and scalability, with the microbial expression systems often being cheaper and easier to scale in comparison with insect or mammalian cell culture setups, although this should always be evaluated on a case by case basis. Expanding our knowledge and toolbox in the field of heterologous toxin expression will hopefully boost the biotechnological applications derived from different subfields in the important area of research that is toxinology.
